# Evaluation of Systemwide Improvement Programs to Optimize Time to Surgery for Patients With Hip Fractures: A Systematic Review

**DOI:** 10.1001/jamanetworkopen.2022.31911

**Published:** 2022-09-16

**Authors:** Pariswi Tewari, Brian F. Sweeney, Jacie L. Lemos, Lauren Shapiro, Michael J. Gardner, Arden M. Morris, Laurence C. Baker, Alex S. Harris, Robin N. Kamal

**Affiliations:** 1 Department of Orthopaedic Surgery, Stanford University, Redwood City, California; 2 Stanford University School of Medicine, Mountain View, California; 3 Department of Orthopaedic Surgery, University of California, San Francisco; 4 Surgery Policy Improvement Research and Education Center, Department of Surgery, Stanford University, Stanford, California; 5 Department of Health Research and Policy, Stanford University, Stanford, California; 6 VOICES Health Policy Research Center, Stanford University, Stanford, California

## Abstract

**Importance:**

Longer time to surgery (TTS) for hip fractures has been associated with higher rates of postoperative complications and mortality. Given that more than 300 000 adults are hospitalized for hip fractures in the United States each year, various improvement programs have been implemented to reduce TTS with variable results, attributed to contextual patient- and system-level factors.

**Objective:**

To catalog TTS improvement programs, identify their results, and categorize program strategies according to Expert Recommendations for Implementing Change (ERIC), highlighting components of successful improvement programs within their associated contexts and seeking to guide health care systems in implementing programs designed to reduce TTS.

**Evidence Review:**

A systematic review was conducted per the Preferred Reporting Items for Systematic Reviews and Meta-analyses guideline. Three databases (MEDLINE/PubMed, EMBASE, and Cochrane Trials) were searched for studies published between 2000 and 2021 that reported on improvement programs for hip fracture TTS. Observational studies in high-income country settings, including patients with surgical, low-impact, nonpathological hip fractures aged 50 years or older, were considered for review. Improvement programs were assessed for their association with decreased TTS, and ERIC strategies were matched to improvement program components.

**Findings:**

Preliminary literature searches yielded 1683 articles, of which 69 articles were included for final analysis. Among the 69 improvement programs, 49 were associated with significantly decreased TTS, and 20 programs did not report significant decreases in TTS. Among 49 successful improvement programs, the 5 most common ERIC strategies were (1) assess for readiness and identify barriers and facilitators, (2) develop a formal implementation blueprint, (3) identify and prepare champions, (4) promote network weaving, and (5) develop resource-sharing agreements.

**Conclusions and Relevance:**

In this systematic review, certain components (eg, identifying barriers and facilitators to program implementation, developing a formal implementation blueprint, preparing intervention champions) are common among improvement programs that were associated with reducing TTS and may inform the approach of hospital systems developing similar programs. Other strategies had mixed results, suggesting local contextual factors (eg, operating room availability) may affect their success. To contextualize the success of a given improvement program across different clinical settings, subsequent investigation must elucidate the association between interventional success and facility-level factors influencing TTS, such as hospital census and type, teaching status, annual surgical volume, and other factors.

## Key Points

**Question:**

What contributes to the success of improvement programs designed to reduce time to surgery (TTS) for adult patients with hip fractures?

**Findings:**

In this systematic review of 69 studies, 49 programs were associated with significant decreases in TTS, and 20 programs were not. Common themes among successful improvement strategies (eg, identifying barriers and facilitators to program implementation) were cataloged according to Expert Recommendations for Implementing Change, and contextual factors contributing to failed experimental results were evaluated.

**Meaning:**

These findings suggest that many of the assessed improvement programs show promise for reducing hip fracture TTS across different clinical settings in the near future.

## Teaser

This systematic review catalogs improvement programs designed to decrease time to surgery for hip fractures, identifies their results, and categorizes program strategies according to the Expert Recommendations for Implementing Change.

## Introduction

Annually, more than 300 000 patients are diagnosed with new hip fractures in the United States, and more than 1.6 million patients present with hip fractures worldwide.^1,2^ The incidence of hip fractures is projected to more than double by 2050,^1,3^ and, given that the geriatric population in the United States is the fastest growing segment of the population, the most rapid growth in annual fracture rates and cost are expected for patients aged between 65 to 74 years.^4^ Hip fractures confer substantial economic burdens to patients and the health care system,^4,5,6,7^ with total annual direct medical costs associated with hip fractures approaching $5.96 billion in the United States.^8,9^ Given that hip fractures are also associated with significant rates of morbidity and mortality, optimizing hip fracture outcomes has, therefore, become a public health priority worldwide.

Among older patients with hip fracture, mortality in the first year following fracture surgery ranges from 15% to 36%, approximately 4 times higher than that of younger patients with hip fractures. The risk for surgical complications and mortality among patients with hip fracture is dependent on multiple factors, including scope and severity of injury, course of treatment, and patient characteristics.^10,11,12^ In particular, a longer time to surgery (TTS) has been identified as an independent risk factor for mortality and surgical complication.^13,14,15^ For example, an operative delay of more than 48 hours has been associated with a 41% increase in the odds of 30-day mortality and as much as 32% increased 1-year mortality.^16^ The impacts of delayed TTS on hip fracture surgical outcomes has therefore motivated work to identify drivers of TTS.^17,18,19,20,21,22^ These drivers include patient-level factors, including socioeconomic status and medication use (eg, anticoagulants), as well as facility-level factors, including hospital payment systems and operating room hours. In response, hospitals have implemented programs dedicated to modifying these factors, particularly at the facility-level scale, with the goal of optimizing TTS and thereby improving patient outcomes.

Across other sectors of medicine, the Expert Recommendations for Implementing Change (ERIC) framework has been used to address barriers to achieving a desired health services outcomes (eg, optimizing response to time-sensitive conditions, such as pulmonary embolism^23^ or acute coronary syndrome^24^) by mapping improvement program strategies. A Cochrane review^25^ found that such tailored improvement can address implementation barriers and improve professional practice and health care outcomes more effectively than untailored strategies. This systematic approach to classifying improvement strategies has not yet been applied to the management of hip fractures. The aims of our study were to (1) catalog improvement programs aimed at reducing TTS and (2) use the ERIC taxonomy to characterize the improvement strategies most commonly used in promising programs. This approach highlights successful improvement program components to provide strategic recommendations for future TTS improvement implementation.

## Methods

### Literature Search Strategy

Our review used the Preferred Reporting Items for Systematic Reviews and Meta-Analysis (PRISMA) methods and was registered with Prospero (CRD42021267021). Two authors (P.T. and J.L.L) conducted literature searches from peer-reviewed journals in 3 medical databases (MEDLINE/PubMed, EMBASE, and Cochrane Trials) to identify improvement programs for which TTS was an experimental outcome. Search strategies included terms such as *hip fracture*, *time to treatment*, *surgery*, and *intervention* (eAppendix 1 in the Supplement). Searches were performed from July 16 to August 13, 2021. Articles published before January 1, 2000, were excluded from screening. Then, search results were filtered according to predetermined inclusion and exclusion criteria (Figure). All eligible publications were uploaded to Covidence, a Cochrane-sanctioned collaborative tool for systematic review screening and data extraction. Duplicate studies were removed.

**Figure.**
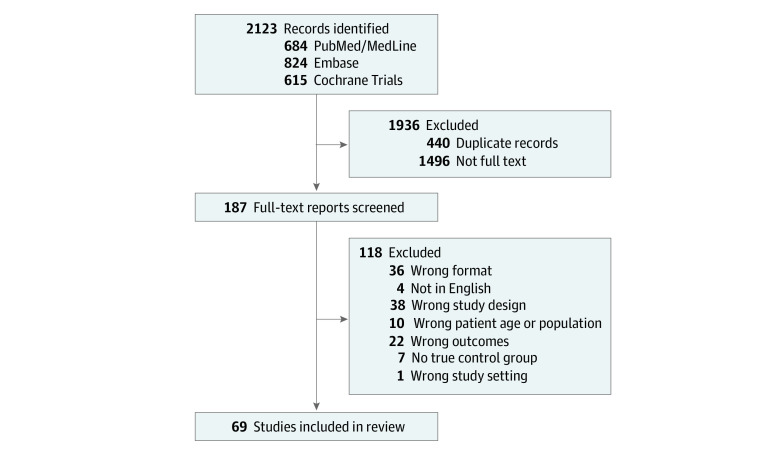
Flow Diagram for Study Selection

### Selection Criteria

This review considered studies that reported on systemwide improvement programs designed to reduce TTS for patients aged 50 years or older, sustaining nonpathological, low-energy hip fractures. Studies that included patients younger than 50 years were excluded. Eligible full-length texts were required to be published or fully translated in English and based in high-income country settings, as defined by the World Bank.^26^ In acknowledgment of wide discrepancies in global health care system resources, we sought to mitigate for confounding variables by selecting improvement programs with comparable high-income country health care settings to the United States. Furthermore, included studies were required to include control and/or baseline data for results on TTS to further elucidate the success of a given improvement program.

Through Covidence, the research team (P.T., B.F.S., and J.L.L.) conducted several stages of text review, consisting of abstract screening, full-text review, and data extraction prior to final article selection. Two researchers independently reviewed eligible texts, according to the predetermined selection criteria, and voted to include or exclude a given text. In the event of a voting conflict, a third reviewer independently resolved disagreements.

### Data Extraction

For each eligible study, data on study design, years of data collection, sample size, improvement program components, results of analysis and variable-outcome associations were extracted onto an Excel spreadsheet and cross-checked for accuracy.

### Quality Appraisal

The 1998 Downs and Black Quality Assessment Checklist^27^ was used to assess the methodological quality of both randomized and nonrandomized studies. This checklist comprises 27 questions across 5 domains: (1) reporting; (2) external validity; (3) internal validity, bias; (4) internal validity, confounding (selection bias); and (5) power. It assigns points to each study accordingly. Studies earning 20 or more points are considered having good individual methodological quality, while studies assigned 14 points or fewer are considered having poor methodological quality and high risk of bias.^28^

### Improvement Program Classification

The ERIC project, introduced by Powell et al^29^ in 2012, maps implementation strategies across studies to mitigate contextual barriers to implementation.^30^ This method, used across other sectors of medicine, creates a standardized and potentially generalizable approach to improvement efforts.^25^ For example, Ebben et al^23^ used ERIC strategies to match improvement program components across studies and optimize emergency department response to time-sensitive conditions such as asthma, pulmonary embolism, triage, acute gastroenteritis, and foot and ankle issues. Similarly, Doucette et al^24^ used ERIC strategies to effectively match improvement program components and improve outcomes for acute coronary syndrome and myocardial injury.

To develop the taxonomy of strategies, extracted data were classified according to 73 ERIC strategies, organized by hierarchical clustering into 9 domain groupings.^31^ The improvement programs identified for our review were partitioned into program components and were independently classified by 2 coauthors (B.F.S. and P.T.) according to concise definitions obtained in literature for each of the 73 ERIC strategies.^32^ Any discrepancies between researchers’ ERIC classifications were discussed and resolved. Descriptive statistics were used to calculate the rate of specific ERIC strategies used among improvement programs that were associated with reduced TTS.

## Results

### Included Studies

A total of 1683 studies were eligible for abstract screening based on literature searches. 190 studies underwent full-text review, and 69 studies were selected for final analysis (Figure and eAppendix 3 in the Supplement).^33,34,35,36,37,38,39,40,41,42,43,44,45,46,47,48,49,50,51,52,53,54,55,56,57,58,59,60,61,62,63,64,65,66,67,68,69,70,71,72,73,74,75,76,77,78,79,80,81,82,83,84,85,86,87,88,89,90,91,92,93,94,95,96,97,98,99,100,101,102,103^ Among the final 69 improvement programs, 49 programs significantly decreased TTS^33,35,38,41,43,44,45,46,47,48,49,51,52,53,54,56,57,59,60,61,62,65,66,67,68,69,70,71,72,74,75,76,79,80,81,82,85,86,87,88,91,94,96,97,99,101,103^ and 20 programs^39,40,42,50,55,58,63,64,73,77,78,83,89,90,92,93,95,98,100,102^ did not. Delayed TTS cutoffs were most often defined dichotomously as more than 24 hours, more than 36 hours, more than 48 hours, or more than 72 hours, although some studies collected continuous data.

### Quality Appraisal

Among the 69 total studies scored, no study earned a checklist score of lower than 20 points, indicating a relatively high-quality body of studies and low risk of bias.^27^ Each study collected and used patient medical records. TTS was also calculated according to hospital medical records and/or databases.

### Determinants of Improvement Program Success

Among the 49 improvement programs associated with significant decreases in TTS, each program had a mean (SD) of 9 (4) strategies defined under ERIC. The 20 improvement programs that did not significantly decrease TTS had a mean (SD) of 8 (2) strategies, from which the 5 improvement programs that were associated with increases in TTS had a mean (SD) of 7 (2) strategies. Successful improvement programs most often had program components corresponding to 3 ERIC domains: (1) use of evaluative and iterative strategies (domain 1; 49/49 studies [100%]); (2) development of stakeholder interrelationships (domain 4; 35/49 studies [71%]); and (3) support of clinicians (domain 6; 33/49 studies [67%]) (Table 1). Specifically, the 5 most common ERIC strategies among improvement programs associated with statistically significant decreases in TTS included assessing for readiness and identifying barriers and facilitators (49 of 49 [100%]), developing a formal implementation blueprint (49 of 49 [100%]), identifying and preparing champions (35 of 49 [71%]), promoting network weaving (35 of 49 [71%]), and developing resource sharing agreements (33 of 49 [67%]) (Table 2). Table 3 lists the recurring ERIC strategy components among unsuccessful programs, and Table 4 presents limitations to those programs.

**Table 1.** Number of ERIC Domains Among Significantly Successful Improvement Programs

Domain title	ERIC domain No.	Occurrence, No. (%) (N = 49)	Example of strategy from literature (source)
Use of evaluative and iterative strategies	1	49 (100)	Develop an implementation blueprint that summarizes the intervention purpose; scope of change; timeframe and intervention milestones; and defines measures of performance and success (Anderson et al,^40^ 2017)
Provision of interactive assistance	2	6 (12.2)	Implement electronic order sets (Anderson et al,^40^ 2017) and e-pathways (Talevski et al,^45^ 2020)
Adapt and tailor to context	3	17 (34.7)	Prioritize surgery of older patients with hip fractures and tailor timing of procedures to within the first hours after admission (Sánchez-Hernández et al,^47^ 2016)
Development of stakeholder interrelationships	4	35 (71.4)	Implement combined multidisciplinary and comanagement systems (VanTienderen et al,^75^ 2021)
Training and education of stakeholders	5	3 (6.1)	Recruit integrated care managers to improve care path compliance and coordination of care (Heyzer et al,^60^ 2021)
Support of clinicians	6	33 (67.3)	Hire a designated lean manager in the orthopaedic department to assess processes involved with quality improvement project, including tracking new hip fracture patients (Sayeed et al,^76^ 2018)
Engagement with consumers	7	1 (2)	In the event that a patient was unable to give consent, delay surgery only when efforts to contact the immediate family failed (Kosy et al,^65^ 2013)
Use of financial strategies	8	6 (12.2)	Incentivize meeting a 24-hour, 48-hour, or other target time for surgical fixation of hip fractures via a reimbursement system (Uri et al,^46^ 2020)
Change of infrastructure	9	10 (20.4)	Designate a dedicated “out of hours” trauma room (Keren et al,^59^ 2017)

Abbreviation: ERIC, Expert Recommendations for Implementing Change.

**Table 2.** Most Common ERIC Strategy Components Among Significantly Successful Improvement Programs

ERIC strategy title	ERIC domain and strategy No.	Occurrence, No. (%) (N = 49)
Assess for readiness and identify barriers and facilitators	1.4	49 (100)
Develop a formal implementation blueprint	1.23	49 (100)
Identify and prepare champions	4.35	35 (71.4)
Promote network weaving	4.52	35 (71.4)
Develop resource sharing agreements	6.30	33 (67.3)
Organize clinician implementation team meetings	4.48	26 (53.1)
Create new clinical teams	6.21	26 (53.1)
Facilitate relay of clinical data to providers	6.32	25 (51.0)
Capture and share local knowledge	4.7	24 (49.0)
Conduct local consensus discussions	4.17	22 (44.9)
Promote adaptability	3.51	17 (34.7)
Conduct local need assessment	1.18	13 (26.5)
Develop an implementation glossary	4.25	11 (22.4)
Change physical structure and equipment	9.11	10 (20.4)
Recruit, designate, and train for leadership	4.57	8 (16.3)
Develop and organize quality monitoring systems	1.27	7 (14.3)
Centralize technical assistance	2.8	6 (12.2)
Provide local technical assistance	2.54	6 (12.2)
Alter incentive and/or allowance structures	8.2	6 (12.2)
Revise professional roles	6.59	6 (12.2)
Use advisory boards and workgroups	4.64	5 (10.2)
Develop and implement tools for quality monitoring	1.26	4 (8.2)
Facilitation	2.33	4 (8.2)
Involve executive boards	4.40	3 (6.1)
Audit and provide feedback	1.5	3 (6.1)
Conduct educational meetings	5.15	3 (6.1)
Inform local opinion leaders	4.38	2 (4.1)
Purposefully reexamine the implementation	1.56	2 (4.1)
Create a learning collaborative	5.20	2 (4.1)
Conduct cyclical small tests of change	1.14	1 (2.0)
Tailor strategies	3.63	1 (2.0)
Obtain formal commitments	4.47	1 (2.0)
Conduct ongoing training	5.19	1 (2.0)
Develop educational materials	5.29	1 (2.0)
Distribute educational materials	5.31	1 (2.0)
Involve patients, consumers, and family members	7.41	1 (2.0)
Conduct educational outreach visits	5.16	1 (2.0)
Model and simulate change	4.45	1 (2.0)
Use an implementation advisor	4.65	1 (2.0)

Abbreviation: ERIC, Expert Recommendations for Implementing Change.

**Table 3.** Recurring ERIC Strategy Components Among Unsuccessful Improvement Programs

ERIC strategy title	ERIC domain and strategy No.	Occurrence, No. (%) (N = 20)
Assess for readiness and identify barriers and facilitators	1.4	20 (100)
Develop a formal implementation blueprint	1.23	17 (85.0)
Develop resource sharing agreements	6.30	16 (80.0)
Identify and prepare champions	4.35	15 (75.0)
Promote network weaving	4.52	14 (70.0)
Organize clinician implementation team meetings	4.48	12 (60.0)
Create new clinical teams	6.21	11 (55.0)
Facilitate relay of clinical data to providers	6.32	11 (55.0)

Abbreviation: ERIC, Expert Recommendations for Implementing Change.

**Table 4.** Limitations Among Unsuccessful TTS Improvement Programs

Top limitations of study design	Top limitations of improvement programs
Participant selection bias (eg, comorbidity rates, relative patient health status, exclusion criteria, small sample sizes)	Multiple improvement programs implemented concurrently (eg, integrated orthogeriatric care with anticoagulant use, comanaged care with multidisciplinary pathways, electronic order sets), making it difficult to discern outcomes of individual improvement strategies
Insufficient system resources (eg, lack of sufficient trauma theaters/beds/staff, lack of complication rates data, among other comprehensive patient data collection insufficiencies, seasonal increases in patient demand by hospital region)	Inadequate specificity of program design (eg, lack of clear documentation for fast-track programs, poor process performance measures, liberal medical recommendations vs conservative delaying TTS rates)
Insufficient leadership (eg, lack of trauma-trained orthopaedic surgeon, lack of geriatrician)	Hesitancy to designate leaders for new clinical teams and follow-up for adherence (eg, hiring staff to oversee program implementation, clearly revising roles and responsibilities within clinical teams)

Abbreviation: TTS, time to surgery.

## Discussion

As a result of the strong body of evidence supporting the association of TTS with morbidity and mortality, various organizations (eg, the American Academy of Orthopaedic Surgeons) have recommended surgical treatment within 48 hours of presentation for patients sustaining hip fractures.^104^ Novel improvement programs have demonstrated potential in reducing in-hospital and 30-day mortality rates as well as decreasing the length of hospital stay.^34,35,36^ Recent studies have further explored the results of improvement programs designed to reduce TTS for hip fracture patients. However, poor consistency in implementation methods has impeded efforts for generalizable strategies for improvement efforts to address hip fracture TTS.^31^ In this systematic review, we cataloged and identified program results using the ERIC classification system. These results highlighted commonalities across studies and provided the foundation for guiding the implementation of future TTS optimization programs in health systems. While designing TTS improvement programs, health care systems may utilize the findings of this review to reference the individual programs we reviewed that bear similarities to the needs of their clinical settings. Additionally, common ERIC strategies and domains of successful programs described in this review can provide nuanced insight into the strategy and design of future TTS improvement programs.

The results of our review indicated that, of the studies included, successful TTS improvement programs had a mean (SD) of 9 (4) strategies defined under ERIC. These studies suggest that a comprehensive strategic approach, using multiple, diverse implementation strategies across different domains, can be beneficial in reducing TTS. Given the studies that did not significantly improve or failed to improve TTS used a mean (SD) of 8 (2) and 7 (2) strategies, respectively, it appears that more implementation strategies were associated with improved TTS. The most prevalent ERIC domains among successful improvement programs were the use of evaluative and iterative strategies (100% rate), development of stakeholder interrelationships (71.4% rate), and support of clinicians (67.3% rate). This pattern suggests the need for increased efficient preoperative communication and partnerships between medical and surgical teams to reduce TTS. Furthermore, the 100% rate of the ERIC strategies assessing for readiness and identify barriers and facilitators and developing a formal implementation blueprint among the 49 successful improvement programs indicates that preemptive strategic planning appears to be positively associated with the success of a given improvement strategy.

The 5 most common ERIC strategies found among improvement programs includes assessing for readiness and identifying barriers and facilitators, describing a tailored, 3-pronged approach to (1) assess the readiness of a given study site to implement an improvement program; (2) assess barriers that could impede implementation efforts; and (3) assess strengths for implementation^31^ (eAppendix 4 in the Supplement). Other common strategies included developing a formal implementation blueprint, which entails an outline of improvement program goals and strategies, such as program aims, scope of organizations affected, timeframe and milestones, and measures of progress and performance. Identifying and preparing champions describes the process of delegating implementation leadership to champions of improvement programs. These champions were often already leaders in the care pathway of the patient and responsible for implementing improvement strategies. Promoting network weaving involves identifying existing networks to (1) promote information sharing and (2) collaborate on solving problems relevant to the improvement program. For example, this strategy often described improvement programs geared toward promoting multidisciplinary and comanaged clinical care. Lastly, developing resource sharing agreements entails developing partnerships with organizations to share resources needed for improvement program implementation. This strategy was also common among improvement programs promoting multidisciplinary and comanaged clinical care.

ERIC strategies common to successful programs were also found among the 20 studies that did not report significant decreases in TTS (Table 3). However, the 5 studies that resulted in increased TTS offered additional, nuanced explanations for the barriers impeding the performance of their improvement programs, including the influence of patient-level factors (eg, anticoagulant use,^42^ confounding comorbidities^63^) (Table 4 and eAppendix 2 in the Supplement). These studies often cited numerous limitations inherent to study design, and relatively sparse limitations to improvement programs, themselves. Nijmeijer et al^42^ cited the introduction of direct oral anticoagulants, which were to be omitted 48 hours prior to surgery, as a potential limitation of its study design and noted that its improvement program defined a lower threshold for delaying surgery to reduce perioperative complications and limited operating room capacity. Lemos et al^89^ supported the potential of dedicated orthopaedic trauma theaters as an improvement program but suggested that a full 7-day theater may optimally decrease TTS, rather than the 4-day theater implemented. This suggestion is consistent with the findings of several other improvement program studies,^35,77,105^ where a consistently designated trauma room achieved lower TTS. Bellas et al^93^ supported the potential of preoperative specialty consultations on hospitalist comanagement as an improvement program but suggested that consultations should be strategically used on a needs-only basis, rather than as standard practice. Finally, the clinical pathway improvement program implemented by Choong et al^55^ included an additional information checklist step in the emergency department, which resulted in a slight increase in TTS that did not yield statistical significance. These 5 studies noted continued barriers to success, including patient-level characteristics or insufficient context-dependent tailoring of strategies to suit the individual needs of each clinical setting. These improvement strategies, including implementing documented protocols for the preoperative care pathway and dedicated operating room times as well as changing surgical prioritization, have demonstrated the potential to be successful at decreasing TTS in some hospital settings, while unsuccessful in others.^74,82^ These conflicting data suggest that both patient-level factors and facility-level factors (eg, hospital census, type, teaching status, annual surgical volume) may affect the success of improvement programs and should be a consideration of future TTS improvement initiatives.

In this review, studies did not commonly include information on facility-level factors when describing the environment of hospital and health care settings. For hospital and health system settings that are already advanced prior to implementation of a given improvement program, smaller margins of program success will be reflected in study results regardless of a given program’s true potential. Therefore, the environment of a health system provides essential context for the performance of a given improvement program. This lack of comprehensive facility-level factor data impeded our ability to provide context for the success of a given improvement program based upon its environment.

Another consideration for our review is the inclusion of studies that only focused on TTS as a main outcome. Several systemwide improvement programs have instead focused on mortality and length of hospital stay as primary outcomes, without also considering changes in TTS. It may be beneficial to consider the replication of these successful programs, with a focus on TTS improvement.

### Limitations

This study has limitations. The ability to gauge the potential of improvement programs was limited by inconsistencies in defining delayed TTS cutoffs between studies. For example, the relative success of an improvement program that reduced TTS to less than 48 hours may depend on the delayed TTS cutoff decided for that study. Similarly, an improvement program aiming to increase surgical intervention within the first 24 hours of patient admission may report smaller margins of program success than an improvement program aiming promote surgical intervention within the first 3 days of patient admission.

It is important to note the heterogeneity of the study age cutoff as a limitation to this review. While geriatric age is typically defined as those 65 years and older, this study included all reports with a patient population aged 50 years and older. This allowed for a more robust analysis of systematic improvements while adding a level of heterogeneity to the study population. Future studies may wish to investigate improvement programs of geriatric populations specifically to identify targeted systematic improvements for this population.

Furthermore, our review was limited by a lack of data regarding program failure, as only 5 included studies described increased TTS results. No failed improvement program provided reasoning for why a program should not be pursued for further implementation efforts in the clinical setting. Thus, the available data informing future improvement program strategies may be limited by publication bias. This publication bias may further occlude research teams from providing reasoning as to why a given improvement program fails and, instead, encourage researchers to focus on cases for future program promise.

Moreover, several studies included TTS data during the improvement program’s implementation as well as after implementation, while other studies merged these data into an overall postimplementation category, limiting an assessment of rates of reduced TTS. Additionally, this review focused on timely surgical management of femoral neck, intertrochanteric, and subtrochanteric fractures only. This was done to minimize confounding considerations for surgical management and to simplify comparisons of improvement program data. Nevertheless, future studies should aim to replicate improvement program results across other hip fracture types to confirm TTS program generalizability. Also, while this systematic review included studies with levels of evidence III and IV, ie controlled trials without randomization and cohort studies, it excluded case reports and case studies to reduce confounding sample sizes; varying sample sizes among our included studies may skew the success rates presented for any given improvement program. Additionally, while each improvement program was independently read and cataloged according to ERIC strategies by 2 authors, and any conflicts were independently reviewed and resolved by a third author, study classifications may be subject to, albeit minimal, manual human error.

## Conclusions

In this review of improvement programs for TTS for hip fracture, we summarized the results of novel programs. Generally, the 69 improvement programs possessed the capability to decrease TTS. Particular promise for replicable success was demonstrated by the 49 improvement programs that were associated with significant TTS decreases. Among these successful programs, general themes were ascertained regarding common ERIC strategies and domains. Future study designs may incorporate these common implementation strategies from previously successful programs to inform and structure novel improvement programs. Finally, this review suggests a need for understanding the facility-level and patient-level factors of potential implementation settings to provide necessary context associated with the success of a given improvement program. By highlighting promising strategies and bodies of work, this review may therefore inform the direction of future TTS improvement programs to promote the development of both generalizable and context-specific strategies for improvement programs in the future.

## Notes

Supplement. **eAppendix 1.** Database Search Criteria **eAppendix 2.** Limitations Among Unsuccessful Improvement Programs **eAppendix 3.** Included Studies, Improvement Programs, Quality Scores, and Results **eAppendix 4.** ERIC Domains and Strategies
