# Supplementary material for: Evaluation of Systemwide Improvement Programs to Optimize Time to Surgery for Patients With Hip Fractures: A Systematic Review
Source: JAMA Netw Open. 2022 Sep 16;5(9):e2231911. doi: 10.1001/jamanetworkopen.2022.31911 (PMC9482052; doi:10.1001/jamanetworkopen.2022.31911)
Supplement: Supplement. — eAppendix 1. Database Search Criteria eAppendix 2. Limitations Among Unsuccessful Improvement Programs eAppendix 3. Included Studies, Improvement Programs, Quality Scores, and Results eAppendix 4. ERIC Domains and Strategies [file jamanetwopen-e2231911-s001.pdf]

Supplemental Online Content

Tewari P, Sweeney BF, Lemos JL, et al. Evaluation of system-wide improvement programs to optimize time to surgery for patients with hip fractures. *JAMA Netw Open.* 2022;5(9):e2231911. doi:10.1001/jamanetworkopen.2022.31911

eAppendix 1. Database Search Criteria

eAppendix 2. Limitations Among Unsuccessful Improvement Programs

eAppendix 3. Included Studies, Improvement Programs, Quality Scores, and Results

eAppendix 4. ERIC Domains and Strategies

This supplemental material has been provided by the authors to give readers additional information about their work.

© 2022 Tewari P et al. *JAMA Network Open.*

eAppendix 1. Database Search Criteria

Database	Search Terms
PubMed/ MEDLINE	(“Hip fracture*”[title] OR “femoral neck fracture*”[tw] OR “hip fractures/surgery”[mesh]) AND (“time to treatment”[mesh] OR “wait time*”[tiab] OR “time to presentation”[tw] OR “time to surgery”[tiab] OR “time management”[mesh] OR “delay time”[tiab] OR “time to theater”[tw] OR “time to surg*”[tiab] OR “timing”[tiab] OR (“delay*”[tiab] AND “surg*”[tiab])) AND (“intervention*”[tw] OR “care pathway*”[tw] OR “pathway*”[tw] OR “comanagement”[tw] OR “system”[tw] OR “outcomes”[tw] OR “improvement*”[tw])
EMBASE	('hip fracture':ti OR (femoral AND neck AND fracture)) AND surgery AND ('time to treatment':ab,ti OR 'wait time':ab,ti OR 'time to presentation':ab,ti OR 'time to surgery':ab,ti OR 'time management':ab,ti OR 'delay time':ab,ti OR 'time to theater':ab,ti OR 'time to surg*':ab,ti OR timing:ab,ti OR (delay*:ab,ti AND surg*:ab,ti)) AND ((intervention* OR care) AND pathway OR pathway OR comanagement OR system OR improvement* OR outcomes)
Cochrane Trials	(hip fracture AND surg*):ti OR (femoral neck fracture AND surg*):ti AND (time to treatment OR wait time OR time to presentation OR time to surgery OR time management OR delay time OR time to theater OR (delay* AND surg*)):ab OR (time to treatment OR wait time OR time to presentation OR time to surgery OR time management OR delay time OR time to theater OR (delay* AND surg*)):ti AND (intervention* OR care pathway OR comanagement):ti,ab,kw

© 2022 Tewari P et al. *JAMA Network Open.*

eAppendix 2. Limitations Among Unsuccessful Improvement Programs

Limitations Among Unsuccessful Improvement Programs				
	Study	Result	Limitations of Study Design	Limitations of Improvement Programs
1	Anderson et al 2017	Decreased TTS - did not achieve statistical significance	Study did not adjust for comorbidities, severity of illness, etc. in patients. Study also could not track ED (Emergency Department) phase of care- no ED care pathway in place.	Multiple concurrent interventions (e.g. comprehensive program included electronic order sets, comanagement, etc.) were implemented- difficult to discern which strategies were effective.
2	Larsson et al 2016	Decreased TTS - did not achieve statistical significance	Selection Bias from missing informed consents and eligible patients that were not included. Attributed to skepticism from ambulance nurses against A&E pathway in favor of PFTC gold standard. High-functioning system: Many control group patients underwent surgery within 24h.	N/A
4	Nijmeijer et al 2018	Did not decrease TTS	Selection Bias- "fittest" elderly were excluded from study and treated with total hip prosthesis. Potential lower threshold for delaying surgery in order to achieve a better medical condition of the patient to reduce the risk of perioperative complications (e.g., in case of a pneumonia). Introduction of the direct oral anticoagulants to patients, which were to be omitted during 48 h before hip fracture surgery can take place	Introduction of the direct oral anticoagulants to patients, which were to be omitted during 48 h before hip fracture surgery can take place.
13	Anighoro et al 2020	Decreased TTS - did not achieve statistical significance	Higher patient volume and comorbidites (CCI) in the postpathway treatment group. Limitations of facility-inpatient bed availability, high acuity patient demand in the emergency department, resource-intensive diagnostic testing (eg, echocardiography), consultant bandwidth, and OR availability. “No divert” policy prevented certain patient transfers to other facilities- may affect patient volume and bed availability.	N/A
18	Choong et al 2000	Did not decrease TTS	Study attempted to alleviate selection bias via included patients with language and cognitive difficulties. Frequent existence of unstable and often untreated pre-morbid conditions in patients with fractured neck of femur, which required attention during acute admission. High-functioning system: had already reduced length of stay for many classes of orthopaedic conditions, including fractured neck of femur. Variation in local factors such as availability of ACAS and support services and patient characteristics.	Referrals for ACAS assessment for placement- these patients stayed significantly longer than patients who did not.
21	Bracey et al 2016	Decreased TTS - did not achieve statistical significance	Small sample sizes. Large number of hip fractures that were excluded from the study- patients were excluded for polytrauma and life-threatening conditions present in addition to a hip fracture. Bias in results- a number of patients meeting initial inclusion criteria were ultimately excluded from the study because they required admission to medical subspecialty services- not eligible for OHC admission and in most cases had significantly longer LOS. Short data collection study design at level 1 trauma center. Inherent limitations of retrospective study design with respect to the pre-OHC group.	N/A
26	Hommel et al 2008	Did not decrease TTS	Lucidity of the participants also markedly influenced reoperation rate and mortality. Both mortality and frequency of reoperation differed significantly with fracture type and surgical method. Intervention group surgery mostly during winter months. Fewer participants suffered administrative operative delay during the summer. Participant ASA grades varied. Intervention group had more comorbidities. Male vs female patient outcomes cited as a limitation. Choice of treatment- healthier<br>people were found suitable for Medoff and intramedullary nail treatment as well as arthroplasty. Control group surgery during summer months- restriction of elective operations and space for acute operations. Half the ward capacity was also closed.	N/A
27	Gholve et al 2005	Decreased TTS - did not achieve statistical significance	Limitation in resources, trial was restricted to the pre-operative phase of the pathway.	35% of the admissions were also inadequately fast tracked- reduced the admission time and skin-related complications, but did not result in early fixation and<br>reduced length of stay in the hospital. Issues with fast tracking- need for clear documentation of every stage of the process and also better communication
37	Kristensen et al 2016	Decreased TTS - did not achieve statistical significance	Potentially higher incidence of comorbidities in study population. Reliability of the data questioned due to collection by a large number of clinicians during routine clinical work and potential for misclassification.	Geriatrician assessed all relevant<br>disorders and disabilities and not only those precipitating the<br>hospital admission- time-consuming. Process performance measures cannot provide information regarding the appropriateness of a patient's treatment
41	McDonald et al 2021	Decreased TTS - did not achieve statistical significance	No trauma-trained orthopaedic surgeon. Potential loss of complications records from patients, as 90-d complications obtained from hospital medical records. PT to OT time was long because physical therapy department had a policy of not seeing patients on the same day as surgery. TTS influenced by hesitancy to schedule after-hours surgery; suspected to cause late-night fatigue.	N/A
42	Rincón Gómez et al 2020	Decreased TTS - did not achieve statistical significance	In the COFRAC cohort, the identification of polypathological patients, of motor deficiency, of arrhythmias other than atrial fibrillation, of osteoporosis and of the number of long-term drugs was higher. Considerable lack of data in the HIST cohort- unable to comparatively analyze variables such as height, weight, functional status, comorbidities (using the Charlson index), the history of falls and the social-familial state. Greater accuracy in the diagnosis of delirium in the COFRAC cohort and to the underestimation of the diagnosis in the HIST cohort. Spanish culture surrounding health- considerable acceptance of care from the family for the elderly and for dependent patients.	Needed standard multidisciplinary discharge protocols in which<br>responsibility is shared among primary care, orthopedic surgeons, internists, physical medicine and rehabilitation.

© 2022 Tewari P et al. *JAMA Network Open.*

	Study	Result	Limitations of Study Design	Limitations of Improvement Programs
48	Marcheix et al 2021	Decreased TTS - did not achieve statistical significance	Could not state criteria for discharge of patients- but only department change during year assessed is the addition of the orthogeriatric unit. Change in habits- more frequent usage of trochanteric nail and spinal anaesthesia in 2018 vs 2017.	N/A
55	Lemos et al 2009	Did not decrease TTS	Choice of impant may have influenced longer wait times- Patients in the PRE group mostly received noncemented vs cemented hip fracture implants. More preoperative comorbidities in POST group. UTI rate x6 higher in PRE vs POST group. Limitation of # of theaters (demand for 6, given 4).	4-day theater, in comparison to 7-day theater, not sufficient to decrease TTS
56	Shenouda et al 2017	Decreased TTS - did not achieve statistical significance	Relatively small patient sample and analysis of patient outcomes only until time of discharge. Patients were more likely to be cancelled on the day of surgery due to inadequate medical optimisation. Most common reason for breeching the 36-hour BPT target for time to surgery was a lack of available morning operating lists, with trauma lists in study hospital unit traditionally scheduled in the afternoon on four out of five days of the working week.	Not all patients with proximal femoral fractures were suitable for this pathway, e.g. polytrauma patients may require a higher level of care.
58	Schuijt et al 2020	Decreased TTS - did not achieve statistical significance	Significantly more missing baseline data and outcome data in the historical cohort. Selection bias in exclusion of younger patients- 69 patients were eligible for inclusion in the study, but were not admitted to the orthogeriatric trauma unit because the unit was at maximum capacity. These patients were younger at baseline than those admitted. Only short-term outcomes were measured in this study. No patient-reported outcome measures or functional outcomes collected.	N/A
59	Bellas et al 2020	Did not decrease TTS	N/A	Designation of minor, moderate, or major recommendation was done after the consults were already completed- may not be possible to predict that a consult results in no change without it being actually performed. Classification of recommendations were somewhat arbitrary and subjective. "unmeasured complexity" noted by the admitting physician. Categorization of consult recommendations and consequent changes by one physician could be considered subjective.
61	Godin et al 2015	Decreased TTS - did not achieve statistical significance	It is likely that no differences in perioperative complications, TTS, or LOS were found when patients admitted to the internal medicine service- only difference in care occurred for those patients who ranged from the middle of the health spectrum to healthy. The patients with more comorbidities and an increased frequency of perioperative complications were already being admitted to the internal medicine team. Patients on the medicine service under the OP had an increased TTS to begin with when compared to those who were admitted to the orthopaedic surgery service. Patients in NP who experienced change in care were already healthier on average. Hospital is not a high-volume hip fracture center and, therefore, study is underpowered.	N/A
65	Pajulammi et al 2017	Decreased TTS - did not achieve statistical significance	During the first years of the orthogeriatric collaboration, the timing of surgery depended on random issues, such as the educational phase of resident orthopedic surgeons, notification of the operation room only after admitting the patient to the orthopedic ward and availability of operation room time. In 2013, Lean model implemented but still not producing significant TTS decreases because a significant change could only be seen in the analysis with time as a continuous factor.	CGA intervention mainly takes place postoperatively and the delay to surgery is not cited as being directly affected by CGA. The lack of leadership from especially the medical physician/geriatrician is considered the greatest barrier for improving hip fracture care.
67	Valsamis et al 2020	Decreased TTS - did not achieve statistical significance	There was a significant decrease in time to surgical intervention during the study period that occurred long before the introduction of the HFU, and that cannot be attributed to the HFU itself. The changes observed in hip fracture patient outcomes over a 6-year period was likely multifactorial."	N/A
69	Shigemoto et al 2019	Decreased TTS - did not achieve statistical significance	For delirium as a postoperative complication, the diagnostic method was ambiguous, so the incidence was expected to be even higher. Lack of smooth cooperation between the departments impeded some TTS data. Was later remedied.	N/A

© 2022 Tewari P et al. *JAMA Network Open.*

eAppendix 3. Included Studies, Improvement Programs, Quality Scores, and Results

© 2022 Tewari P et al. *JAMA Network Open.*

Study		Improvement Program	Quality Score	Result	Significance	Worked? Y/N
Anderson 2017	Geriatric Hip Fracture Care: Fixing a Fragmented System.	Comprehensive geriatric hip fracture program	3	Improvement program decreased TTS from 29.0 to 26.5 hours (p = 0.168).	Not Significant	Y
Larsson 2016	Prehospital fast track care for patients with hip fracture: Impact on time to surgery, hospital stay, post-operative complications and mortality a randomised, controlled trial.	Pre-hospital (prior to accident and emergency ward) fast track care	1	improvement program either decreased or had no effect on TTS- mean reduction of 1.85 hours (P = 0.07) (95% CI: −0.12 to 3.82). No difference in times between start of X-ray to start of surgery (Table 2, Fig. 4). No significant differences in surgery within 24 h	Not Significant	Y
Miura 2009	Effects of a Geriatrician-Led Hip Fracture Program: Improvements in Clinical and Economic Outcomes: Geriatrician-Led Hip Fracture Service Outcomes	Geriatrician-led Hip Fracture Service (HFS)	2	Improvement program significantly decreased TTS- The number of patients that went to surgery in less than 24 hours went from 22.2% to 50.5%, between 24 and 36 hours went from 52.8% to 41.8%, between 36 and 48 hours went from 16.7% to 3.3%, and longer than 48 hours went from 8.3% to 4.4%. (P=0.001)	Significant	Y
Nijmeijer 2018	The consistency of care for older patients with a hip fracture: are the results of the integrated orthogeriatric treatment model of the Centre of Geriatric Traumatology consistent 10 years after implementation?	Centre for Geriatric Traumatology (CvGT)- Integrated orthogeriatric treatment model	2	Improvement program did not decrease TTS- rate of surgery within 24 h after arrival to the hospital decreased (P1 72.9%, P2 73.8%, P3 64.7%, p < 0.001), whereas surgery within 48 h after arrival to the hospital did not change over the periods (p 0.085)	Significant, but in opposite direction	N
O'Mara-Gardner 2020	Establishing a Geriatric Hip Fracture Program at a Level 1 Community Trauma Center.	Geriatric hip fracture program (GFP)	3	Improvement program significantly decreased TTS- The number of patients who underwent surgery within 24 hours of admission went from 42.2% to 67.2% (p < .0001).	Significant	Y
Pablos-Hernandez 2020	Time to Surgery Reduction in Hip Fracture Patients on an Integrated Orthogeriatric Unit: A Comparative Study of Three Healthcare Models	Geriatric Consultant Model (GCM)- integrated orthogeriatric unit	3	Improvement program significantly decreased TTS- Rate of patients who underwent surgery in the first 24 h after admission went from 5% to about 6.6% in the geriatric consult model, or to about 25% in the orthogeriatric unit model. (P < 0.001).	Significant	Y
Talevski 2020	Implementation of an electronic care pathway for hip fracture patients: a pilot before and after study.	Electronic care pathway	3	Improvement program significantly decreased TTS- patients undergoing surgery greater than 48 hours after admission went from 77 patients (59.7%) to 11 patients (21.6%); unadjusted (OR = 0.19; CI 0.09–0.39; p < 0.001) and adjusted (OR = 0.22; CI 0.10–0.49; p < 0.001) models	Significant	Y
Uri 2020	A Reimbursement System Based on a 48-Hour Target Time for Surgery Shortens the Waiting Time for Hip Fracture Fixation in Elderly Patients.	Reimbursement system incentivizing meeting a 48-hour target time for surgical fixation of hip fractures	2	Improvement program significantly decreased TTS- average TTS went from 77 hours to 28 hours (P= 0.001). Surgeries delayed beyond 48 hours went from 41% to 20% (P= 0.001).	Significant	Y
Sánchez-Hernández 2016	Results following the implementation of a clinical pathway in the process of care for elderly patients with osteoporotic hip fracture in a second level hospital	12-step clinical pathway for hip fracture care	2	Improvement program significantly decreased TTS- A decrease of 29.4% was observed (from 6.23 to 4.40 days, P = 0.000)	Significant	Y
Noticewala 2016	Multidisciplinary care of the hip fracture patient: a case control analysis of differing treatment protocols	Combined multidisciplinary service	3	Improvement program significantly decreased TTS- from 2.4 (± 2.2) days to surgery to 1.7 (± 1.8) days to surgery	Significant	Y
Kusen 2021	Different approaches towards geriatric trauma care for hip fracture patients: an inter-hospital comparison	Geriatric care pathway	3	Improvement program significantly decreased TTS- number of patients undergoing surgery within 24 hours went from 124 (32.2%) to 95 (18.5%) (p<0.0001), within 48 hours went from 212 (55.1%) to 320 (62.4%) (p=0.028), within 72 hours went from 49 (12.7%) to 98 (19.1%) (p 0.011)	Significant	Y
Chechik 2012	In support of early surgery for hip fractures sustained by elderly patients taking clopidogrel: a retrospective study.	Differential timing of hip fracture surgical intervention for patients taking blood thinner medication	3	Improvement program significantly decreased TTS- from 7 days and 12 hours – 2 days and 17 hours (mean-SD) to 1 day and 16 hours – 1 day [p < 0.0001]	Significant	Y
Anigboro 2020	From ER to OR: Results After Implementation of Multidisciplinary Pathway for Fragility Hip Fractures at a Level 1 Trauma Center.	Multidisciplinary care pathway	3	Improvement program decreased TTS- Average TTS was 0.89 days (pre) versus 0.75 days (post) (P ¼ .20). The percentage of patients receiving surgery in <24 hours was 63.8% (pre) and 72.8% (post) (P ¼ .12); the percentage of patients receiving surgery in <48 hours was 92.2% (pre) and 93.2% (post) (P ¼ 0.77; Table 1).	Not Significant	Y
Biber 2013	Implementation of a co-managed Geriatric Fracture Center reduces hospital stay and time-to-operation in elderly femoral neck fracture patients.	Geriatric Fracture Center "N-Active" orthogeriatric ward	3	Improvement program significantly decreased TTS- TTS decreased from 3.1 to 2.1 days (p = 0.029).	Significant	Y
Roy 2006	Associations between the hospitalist model of care and quality-of-care-related outcomes in patients undergoing hip fracture surgery.	Hospitalist consultant model of care	3	Improvement program significantly decreased TTS- patients receiving surgery within 24 hours of admission went from 11% (5-21) to 32% (19-47) (P=.004)	Significant	Y
Metcalfe 2019	Pay for performance and hip fracture outcomes: an interrupted time series and difference-in-differences analysis in England and Scotland.	Performance-based payment model for hip fracture outcomes	4	Improvement program significantly decreased TTS- from patients undergoing surgery within 36 hours (annual trend -0.6 percentage-points; 95% CI -0.9 to -0.4). to 15.4 percentage-points (95% CI 13.7 to 17.0; Table II) and increasing 0.7 percentage-points (95% CI 0.5 to 0.8) each year thereafter	Significant	Y
Divecha 2011	Improving patient flow: the impact of consultant work pattern on trauma ward efficiency.	Multidisciplinary trauma care pathway	3	Improvement program significantly decreased TTS- the percentage of patients who went to theatre within 24 h of admission increased from 60% before the rota change to 78% after the rota change (p < 0.001).	Significant	Y
Choong 2000	Clinical pathway for fractured neck of femur: a prospective, controlled study.	Multidisciplinary clinical pathway	2	Improvement program did not decrease TTS- TTS increased from 16.9 Hours to 17.5 hours (p=0.72)	Not Significant	N
Elder 2005	The effectiveness of orthopaedic trauma theatres in decreasing morbidity and mortality: a study of 701 displaced subcapital hip fractures in two trauma centres.	Dedicated Trauma OR	3	Improvement program significantly decreased TTS- TTS went from 56.5 h to 29.3 h. (p < 0.0001)	Significant	Y
Bohm 2015	Reduced time to surgery improves mortality and length of stay following hip fracture: results from an intervention study in a Canadian health authority.	Interventions to meet a 48-hour benchmark for hip fracture surgery	3	Improvement program significantly decreased TTS- The proportion of patients receiving surgery within 48 hours increased from 66.8% to 84.6% (p < 0.001)	Significant	Y
Bracey 2016	An Orthopedic-Hospitalist Comanaged Hip Fracture Service Reduces Inpatient Length of Stay.	Combined orthopedic-hospitalist comanaged (OHC) hip fracture service	2	Improvement program decreased TTS- went from 27.4 to 21.9 hours (P ¼ .27) and surgery within 48 hours increased from 86% to 96% (P ¼ .15).	Not Significant	Y
Keren 2017	The effect of 'Out of hours surgery Service' in Israel on hip fracture fixation outcomes: a retrospective analysis.	'Out of Hours Surgery Service' (OHSS) system	3	Improvement program significantly decreased TTS- 42% vs. 33% of patients were operated within the first 24 h, and 38% vs. 20% within 24 to 48 h respectively, with a p-value of < 0.0001)	Significant	Y
Heyzer 2021	Integrated Hip Fracture Care Pathway (IHFCP): reducing complications and improving outcomes.	Integrated Hip Fracture Care Pathway (IHFCP)	2	Improvement program significantly decreased TTS- surgeries performed within 48 hours of admission went from 32.5% to 80.1%	Significant	Y
Kalmet 2016	Effectiveness of a Multidisciplinary Clinical Pathway for Elderly Patients With Hip Fracture: A Multicenter Comparative Cohort Study.	Multidisciplinary Clinical Pathway	3	Improvement program significantly decreased TTS- TTS went from 24.4 (22.5) hours to 19.2 (23.5) hours (P < .01). The number of patients who had to wait more than 24 hours also went from 35% to 23% (P < .01)	Significant	Y
Khoriati 2014	Does a Dedicated Unit for the Treatment of Hip Fractures Improve Acute Outcomes?	Specialist hip fracture unit	3	Improvement program significantly decreased TTS- went from 34 hours to 28 hours (P = 0.04).	Significant	Y
Hommel 2008	Influence of optimised treatment of people with hip fracture on time to operation, length of hospital stay, reoperations and mortality within 1 year.	Documented protocol for rapid transition from the acute emergency ward to the ordinary orthopaedic ward	2	Improvement program did not decrease TTS- The total mean waiting time from admission to operation increased from 26.5 (S.D. 23.6) h to 27.4 (S.D. 16.3) h in	Significant, in opposite direction	N
Gholve 2005	Multidisciplinary integrated care pathway for fractured neck of femur. A prospective trial with improved outcome.	Multidisciplinary integrated care pathway (ICP) programme	2	Improvement program did not decrease TTS- went from 79% (105/ 133) undergoing surgery within 24 h of admission compared to 71% (108/143)(P < 0.05).	Not Significant	Y
Kosy 2013	Fractured neck of femur patient care improved by simulated fast-track system.	Fast track management system	2	Improvement program significantly decreased TTS- The mean time to theatre initially reduced from 44.95 (±27.42) to 29.25 (±21.23) hrs (P 0.001).	Significant	Y
Gilchrist 2017	Enhanced hip fracture management: use of statistical methods and dataset to evaluate a fractured neck of femur fast track pathway-pilot study.	Fracture Neck of Femur Fast Track Pathway and weekly analytical dataset, 'signalfromnoise' (SFN)	2	Improvement program significantly decreased TTS- The average wait for theatre decreased by 3.6 hours (p=0.044) from 1.62 days to 1.47 days	Significant	Y
Haugan 2017	Mortality and readmission following hip fracture surgery: a retrospective study comparing conventional and fast-track care.	Fast-track care	3	Improvement program significantly decreased TTS- from 31.2 (25.1) hrs to 25.2 (21.2) hours (p<0.001)	Significant	Y
Kalmet PHS 2019	Long-term Patient-reported Quality of Life and Pain After a Multidisciplinary Clinical Pathway for Elderly Patients With Hip Fracture: A Retrospective Comparative Cohort Study.	Multidisciplinary team and change in value priority	3	Improvement program significantly decreased TTS- The mean TTS went from 25.3 hrs (13.9) to 18.2 (9.3) hours (P < .01). The number of patients who had to wait more than 24 hours went from 44.9% to 17.6% (P < .01).	Significant	Y
Baroni 2019	The orthogeriatric comanagement improves clinical outcomes of hip fracture in older adults.	Dual intervention strategy assessment- Orthogeriatric comanagement (OGC) vs Orthopedic team with the support of a geriatric consultant service (GCS)	4	Improvement program significantly decreased TTS- TTS went from 4.5 ± 3.8 days to 3.3 ± 2.1 in orthogeriatric comanagement or to 4.2 ± 2.3 days in geriatric consultation service (p= 0.034). TTS < 48 h also went from 58% to 47% in orthogeriatric comanagement or to 19% in geriatric consultation service (p< 0.001)	Significant	Y
Collinge 2013	Measures of clinical outcome before, during, and after implementation of a comprehensive geriatric hip fracture program: is there a learning curve?	Comprehensive hip fracture program	3	Improvement program significantly decreased TTS- from 51.0 hrs (range, 6–338) to 39.2 hrs(range, 7–269) (p=0.007)	Significant	Y
Colais 2013	The impact of a pay-for-performance system on timing to hip fracture surgery: experience from the Lazio Region (Italy).	Pay-for-performance model based on quality of health care	3	Improvement program significantly decreased TTS- The share of patients with hip fracture that had surgery within 48 hours went from 11.7% to 22.2%. The proportion of early hip fracture operations increased after the pay-for-performance act, regardless of hospital payment type. The largest increase of surgery within 48 h occurred in private hospitals (p < 0.001).	Significant	Y
Yuan 2019	Management of elderly hip fractures by an orthopaedic trauma surgeon reduces surgical delays but does not improve outcomes compared to non-trauma surgeons.	Established orthogeriatric co-managed hip fracture care pathway	3	Improvement program significantly decreased TTS- patients undergoing surgery within 48 h went from 30.5% to 37.5% (p=0.039) patients undergoing surgery within 24 h went from 8% to 9.3% (p=0.507)	Significant	Y
Diament 2015	"Early Trigger" Intravenous Vitamin K: Optimizing Target-Driven Care in Warfarinised Patients With Hip Fracture.	Best practice tariff (BPT) financial incentive model	3	Improvement program significantly decreased TTS- Time to theater went from 53.71 (1.7-128) hours to 37.61 (14.73-71.75) hours (p<.0001)	Significant	Y
Kristensen 2016	Can improved quality of care explain the success of orthogeriatric units? A population-based cohort study.	Geriatrician-led multidisciplinary orthogeriatric unit	2	Improvement program decreased TTS- went from 23.4 hrs to 22.0 hrs (95% CI 0.89–1.26).	Not Significant	Y
Reguant 2019	Efficacy of a multidisciplinary approach on postoperative morbidity and mortality of elderly patients with hip fracture.	Multidisciplinary Hip Fracture Unit (HFU)	2	Improvement program significantly decreased TTS- Surgery performed within 48 h of admission went from 38.3% to 55.1% (p < 0.001).	Significant	Y
VanTienderen 2021	Implementation of a Multidisciplinary "Code Hip" Protocol Is Associated with Decreased Time to Surgery and Improved Patient Outcomes	"Code Hip" protocol	3	Improvement program significantly decreased TTS- Time from presentation to surgery went from 33.2 ± 27.2 hours to 23.1 ± 16.4 hours (p< 0.001)	Significant	Y
Sayeed 2018	Implementation of a Hip Fracture Care Pathway Using Lean Six Sigma Methodology in a Level I Trauma Center.	Lean Six Sigma Methodology-based hip fracture care pathway	3	Improvement program significantly decreased TTS- The percentage of patients who received surgical fixation beyond 48 hours significantly decreased (went from 9.50% to 4.23%; P = 0.01).	Significant	Y
McDonald 2021	Effect of a 6 am-9 am Dedicated Orthopaedic Trauma Room on Hip Fracture Outcomes in a Community Level II Trauma Center.	6 AM–9 AM Weekday Dedicated Orthopaedic Trauma Room	3	Improvement program had mixed effects on TTS- the median TTS went from 27.7 and 27.5 hours. When excluding Friday and Saturday patients, median TTS actually increased non-significantly from 27.1 hours to 27.4 hours. The median TTS for after hours patients was 28.3 hours vs. patients treated during the daytime had a time of 27.0 hours.	Not Significant	Mixed results
Rincón Gómez 2020	Hip fracture co-management in the elderly in a tertiary referral hospital: A cohorts study.	Co-management system	3	Improvement program decreased TTS- went from 3.01 +/- 2.85 days to surgery to 2.93 +/- 2.28 days to surgery	Not Significant	Y
Kommer 2014	Changing the consultant on calls from a daily to weekly rotation system reduces time to theater for patients with hip fracture to improve quality of care: a retrospective study of 2 cohorts of patients presenting with hip fracture.	Weekly rotation of consultants-on-call instead of daily rotational system	3	Improvement program significantly decreased TTS- average time to theater went from 44 hours 46 minutes to 32 hours 19 minutes. Number of patients receiving surgery within 36 hours went from 44.7% to 71.7%. (p= 0.0119)	Significant	Y
Mittal 2018	ValuedCare program: a population health model for the delivery of evidence-based care across care continuum for hip fracture patients in Eastern Singapore.	ValuedCare (VC) Hip Fracture Program	2	Improvement program significantly decreased TTS- surgical treatment within 48 h from admission went from 18.8% to 48%. Median TTS was reduced from 97 to 50.5 hrs.	Significant	Y
Rostagno 2016	In Hospital and 3-Month Mortality and Functional Recovery Rate in Patients Treated for Hip Fracture by a Multidisciplinary Team.	Multidisciplinary Working Group- hip fracture care unit	2	Improvement program significantly decreased TTS- patients treated within 48 hours went from 26% to 54% (P<0.0001)	Significant	Y
Khasraghi 2005	Effectiveness of a multidisciplinary team approach to hip fracture management.	Multidisciplinary hip fracture service	3	Improvement program significantly decreased TTS- patients receiving surgery in <24 hrs went from 84 (35%) to 172 (63%). Patients receiving surgery between 24-48 hours went from 80 (34%) to 63 (23%) and patients reiving surgery >48 hours went from 73 (31%) to 38 (14%).	Significant	Y
Roberts 2021	Protocol-based interdisciplinary co-management for hip fracture care: 3 years of experience at an academic medical center.	Interdisciplinary four-pronged co-management approach	3	Improvement program significantly decreased TTS- went from 37.0±47.7 hrs to 28.5±27.1 hrs (p=0.0016)	Significant	Y
Marcheix 2021	Impact of orthogeriatric management on the average length of stay of patients aged over seventy five years admitted to hospital after hip fractures.	Geriatrician-led orthogeriatric unit	3	Improvement program mostly decreased TTS-patients receiving surgery within 24 hrs went from 13 (5.3%) vs 21 (7.3%) (p=0.62), patients receiving surgery from 24- 48 hrs post-admission went from 75 (30.5%) vs. 80 (27.8%) and patients receiving surgery longer than 48 hrs post-admission went from 158 (64.2%) to 187 (65%)	Not Significant	Y
Tittel 2020	Clinical pathways for geriatric patients with proximal femoral fracture improve process and outcome.	Multidisciplinary clinical pathways	2	Improvement program significantly decreased TTS for HR group: for HR: Hip Replacement group, TTS went from 39.3 hours to 20.6 hrs (p = 0.025); for Open Reduction Internal Fixation group, TTS went from 16.3 hours to 14.3 hours (p = 0.219);	Significant for Hip Replacement group	Y
Middleton 2017	Improving hip fracture outcomes with integrated orthogeriatric care: a comparison between two accepted orthogeriatric models.	Integrated orthogeriatric care	3	Improvement program significantly decreased TTS- went from 41.8 to 27.2 hrs (P < 0.001)	Significant	Y
Kohli 2019	A hip fracture nurse specialist has a positive outcome on the length of stay for patients with hip fractures.	Clinical Nurse Specialist-led multi-disciplinary team	3	Improvement program significantly decreased TTS- went from 1.38 days to 1.15 days (p=0.0001).	Significant	Y
Jackson 2019	Comprehensive Hip Fracture Care Program: Successive Implementation in 3 Hospitals.	Comprehensive Hip Fracture Care Program (HFCP)	3	Improvement program significantly decreased TTS- Mean (SD) time from admission to the operating room decreased from 30.8 (21.1) to 25.6 (20.5) hours (mean difference 5.2 hours; P < .05).	Significant	Y
Sermon 2019	The implementation of a clinical pathway enhancing early surgery for geriatric hip fractures: how to maintain a success story?	Clinical pathway based on monthly system threshold for time to surgery within 48 hours	2	Improvement program significantly decreased TTS- went from 37.3 hrs to 18.7 hrs (P <0.001).	Significant	Y
Phy 2005	Effects of a hospitalist model on elderly patients with hip fracture.	Multidisciplinary hospitalist service	3	Improvement program significantly decreased TTS- mean (SD) TTS went from 38 [47] hours to 25 [53] hours; P<.001)	Significant	Y
Lemos 2009	Dedicated orthopedic trauma theatres: effect on morbidity and mortality in a single trauma centre.	Dedicated orthopedic trauma center	3	Improvement program did not decrease TTS- The operative delay from time from triage in the emergency department until the start of surgery went from in the 56.5 hours to 72.1 hours (p = 0.006)	Significant, in opposite direction	N
Shenouda 2017	The Introduction of a Multidisciplinary Hip Fracture Pathway to Optimise Patient Care and Reduce Mortality: A Prospective Audit of 161 Patients.	Multidisciplinary hip fracture pathway	2	Improvement program decreased TTS- went from 37 hours to 31 hours (p= 0.0663)	Not Significant	Y
Burton 2020	A Multidisciplinary Approach to Expedite Surgical Hip Fracture Care.	Multidisciplinary geriatric hip fracture patient care pathway	3	Improvement program significantly decreased TTS- average TTS went from 20.22 hours (1.5-119.7) to 15.33 (1.4-109.8) hours (P ¼ .01) in 2014 and further decreased to 14.63 (1.4-54.5) hours in 2015 (P ¼ .00). Percent of patients who made it to the operating room within 24 hours went from 74.67% in 2013, to 82.31% in 2014 (P ¼ .10), and then to 84.14% in 2015 (P ¼ .04).	Significant (for Pre to Post- continous and categorical)	Y
Schuijt 2020	Orthogeriatric Trauma Unit Improves Patient Outcomes in Geriatric Hip Fracture Patients.	Orthogeriatric Trauma Unit care pathway	3	Improvement program decreased TTS- TTS went from 21 (16-25) hours to 20 (15-25) hours (p= 0.343*)	Not Significant	Y
Bellas 2020	Impact of Preoperative Specialty Consults on Hospitalist Comanagement of Hip Fracture Patients.	Impact of preoperative specialty consult on hospitalist comanagement system	3	Improvement program did not decrease TTS- The median TTS went from 22.1 hours to 34.3 hours. The percentage of patients receiving surgery within 24 hours went from 58.6% to 23.7% and within 48 hours went from 90.1% to 76.8%.	Significant, in opposite direction	N
Blauth 2021	Geriatric fracture centre vs usual care after proximal femur fracture in older patients: what are the benefits? Results of a large international prospective multicentre study.	Geriatric fracture centre (GFC)	2	Improvement program significantly decreased TTS- Time from hospital admission to surgery (hours) went from 72.0 (77.8) to 33.9 (26.0) hrs (p<0.001*)	Significant	Y
Godin 2015	Two admission pathways for elderly patients with hip fracture: Clinical outcomes at a single institution	Direct admission protocol to internal medicine service with consults from orthopaedic surgery service	3	Improvement program decreased TTS for the orthopaedic surgery service- went from 27.02 hours to 20.72 ± 21.27 hrs for the orthopaedic surgery service (P < 0.0001); went from 27.02 hours to 31.13 ± 33.12 hrs for the medicine service (95% CI: 25.17-37.09)	Not Significant	Y
Hormaechea Bolado 2021	Development and implementation of a perioperative management guide for patients with hip fracture: Health management and clinical impact.	Intrahospital clinical guidelines for perioperative patient management in geriatric fracture centre	2	Improvement program significantly decreased TTS- percent of patients undergoing surgical Improvement program in under 48 hrs went from 8.43% to 39.22% (p= 0.001)	Significant	Y
Kusen 2019	Different approaches towards geriatric trauma care for hip fracture patients: an inter-hospital comparison.	Multidimensional interdisciplinary geriatric trauma care pathway	3	Improvement program significantly decreased TTS- surgical Improvement program at day of admission went from 95 (18.5%) to 124 (32.2%); (p< 0.0001)	Significant	Y
McNamara 2014	Use of lean principals to improve flow of patients with fractured neck of femur—the HOPE study.	Fast-track management protocol and multidisciplinary care pathway	2	Improvement program significantly decreased TTS- The percentage of patients who received surgery within 24 hours went from 44% to 56%, between 24-48 hours went from 21% to 17% and greater than 48 hrs went from 35% to 27%	Significant	Y
Pajulammi 2017	Association of comprehensive geriatric assessment with quality-related care practices during implementation and development of an orthogeriatric hip fracture program	Hip fracture program (HFP) in orthopedic ward	2	Improvement program decreased TTS- The percentage of patients who received surgery within 24 hours stayed at 42% before/after Improvement program, between 24-47 hours went from 41% to 42% and greater than 47 hrs went from 17% to 16% (p=0.923)	Not Significant	Y
Pinnarelli 2012	What drives hospital performance? The impact of comparative outcome evaluation of patients admitted for hip fracture in two Italian regions.	DRG (Diagnosis-Related Group) reimbursement rate for hospital's performance.	2	Improvement program significantly decreased TTS- The proportion of hip operations performed within 48 h was increased by 34% for Lazio (p<0.001) and 46% for Tuscany (p<0.001) and reduced by 3% in other Italian regions (p<0.001).	Significant	Y
Valsamis 2020	Modelling the effect of a dedicated hip fracture unit on patient outcomes using segmented robust linear regression techniques.	Hip Fracture Unit	3	Improvement program decreased TTS- went from 21.51 hours to 20.75 hours (p = 0.150)	Not Significant	Y
Wu 2019	The effect of a multidisciplinary co-management program for the older hip fracture patients in Beijing: a "pre- and post-" retrospective study.	Multidisciplinary co-management care plan	3	Improvement program significantly decreased TTS- The proportion of patients receiving surgery within 48 h after admission went from 6.4% to nearly 50% in the Improvement program group (P < 0.0001).	Significant	Y
Shigemoto 2019	The effect of a multidisciplinary approach on geriatric hip fractures in Japan.	In-hospital multi-pronged intervention	2	Improvement program decreased TTS- The rates for patients who received surgery within 48 h went from 72.5% to 75.2%. Average TTS was reduced by 3 h 7 min	Not Significant	Y

© 2022 Tewari P et al. *JAMA Network Open.*

eAppendix 4. ERIC Domains and Strategies

1. use of evaluative and iterative strategies (10)
    a. 4: Assess for readiness and identify barriers and facilitators
        i. Definition: Assess various aspects of an organization to determine its degree of readiness to implement, barriers that may impede implementation, and strengths that can be used in the implementation effort.
    b. 5: Audit and provide feedback
        i. Definition: Collect and summarize clinical performance data over a specified time period and give it to clinicians and administrators to monitor, evaluate, and modify provider behavior.
    c. 14: Conduct cyclical small tests of change
        i. Definition: Implement changes in a cyclical fashion using small tests of change before taking changes system-wide. Tests of change benefit from systematic measurement, and results of the tests of change are studied for insights on how to do better. This process continues serially over time, and refinement is added with each cycle.
    d. 18: Conduct local need assessment
        i. Definition: Collect and analyze data related to the need for the innovation.
            1. Look for: Pre-surveys
    e. 23: Develop a formal implementation blueprint
        i. Definition: Develop a formal implementation blueprint that includes all goals and strategies. The blueprint should include: 1) aim/purpose of the implementation; 2) scope of the change (e.g., what organizational units are affected); 3) timeframe and milestones; and 4) appropriate performance/progress measures. Use and update this plan to guide the implementation effort over time.
    f. 26: Develop and implement tools for quality monitoring
        i. Definition: Develop, test, and introduce into quality-monitoring systems the right input—the appropriate language, protocols, algorithms, standards, and measures (of processes, patient/consumer outcomes, and implementation outcomes) that are often specific to the innovation being implemented.
            1. Look for: routine assessments, measures, etc.
    g. 27: Develop and organize quality monitoring systems
        i. Definition: Develop and organize systems and procedures that monitor clinical processes and/or outcomes for the purpose of quality assurance and improvement.
    h. 46: Obtain and use patients/consumers and family feedback
        i. Definition: Develop strategies to increase patient/consumer and family feedback on the implementation effort.
    i. 56: Purposefully reexamine the implementation

© 2022 Tewari P et al. *JAMA Network Open.*

        i. Definition: Monitor progress and adjust clinical practices and implementation strategies to continuously improve the quality of care.
    j. 61: Stage implementation scale up
        i. Definition: Phase implementation efforts by starting with small pilots or demonstration projects and gradually moving to a system wide rollout.

2. <u>provision of interactive assistance (4)</u>
    a. 8: Centralize technical assistance
        i. Definition: Develop and use a centralized system to deliver technical assistance focused on implementation issues.
            1. Look for: ERIC #6.32 + technical component
    b. 33: Facilitation
        i. Definition: A process of interactive problem solving and support that occurs in a context of a recognized need for improvement and a supportive interpersonal relationship.
    c. 53: Provide clinical supervision
        i. Definition: Provide clinicians with ongoing supervision focusing on the innovation. Provide training for clinical supervisors who will supervise clinicians who provide the innovation.
    d. 54: Provide local technical assistance
        i. Definition: Develop and use a system to deliver technical assistance focused on implementation issues using local personnel.
            1. Look for: local personnel setting up the tech stuff

3. <u>adapt and tailor to context (4)</u>
    a. 51: Promote adaptability
        i. Definition: Identify the ways a clinical innovation can be tailored to meet local needs and clarify which elements of the innovation must be maintained to preserve fidelity.
    b. 63: Tailor strategies
        i. Definition: Tailor the implementation strategies to address barriers and leverage facilitators that were identified through earlier data collection.
    c. 67: Use data experts
        i. Definition: Involve, hire, and/or consult experts to inform management on the use of data generated by implementation efforts.
    d. 68: Use data warehousing techniques
        i. Definition: Integrate clinical records across facilities and organizations to facilitate implementation across systems.

4. <u>development of stakeholder inter-relationships (17)</u>

© 2022 Tewari P et al. *JAMA Network Open.*

a. 6: Build a coalition
    i. Definition: Recruit and cultivate relationships with partners in the implementation effort.
b. 7: Capture and share local knowledge
    i. Definition: Capture local knowledge from implementation sites on how implementers and clinicians made something work in their setting and then share it with other sites.
        1. Look for: Specific written methods section on local knowledge collection
c. 17: Conduct local consensus discussions
    i. Definition: Include local providers and other stakeholders in discussions that address whether the chosen problem is important and whether the clinical innovation to address it is appropriate.
        1. Look for: mentions of stakeholders, external partners in building implementation
d. 24: Develop academic partnerships
    i. Definition: Partner with a university or academic unit for the purposes of shared training and bringing research skills to an implementation project.
e. 25: Develop an implementation glossary
    i. Definition: Develop and distribute a list of terms describing the innovation, implementation, and the stakeholders in the organizational change.
f. 35: Identify and prepare champions
    i. Definition: Identify and prepare individuals who dedicate themselves to supporting, marketing, and driving through an implementation, overcoming indifference or resistance that the intervention may provoke in an organization.
        1. Look for: Leadership designations
g. 36: Identify early adopters
    i. Definition: Identify early adopters at the local site to learn from their experiences with the practice innovation.
h. 38: Inform local opinion leaders
    i. Definition: Inform providers identified by colleagues as opinion leaders or ‘educationally influential’ about the clinical innovation in the hopes that they will influence colleagues to adopt it.
i. 40: Involve executive boards
    i. Definition: Involve existing governing structures (e.g., boards of directors, medical staff boards of governance) in the implementation effort, including the review of data on implementation processes.
j. 45: Model and simulate change
    i. Definition: Model or simulate the change that will be implemented prior to implementation.
k. 47: Obtain formal commitments
    i. Definition: Obtain written commitments from key partners that state what they will do to implement the innovation.
l. 48: Organize clinician implementation team meetings

        i. Definition: Develop and support teams of clinicians who are implementing the innovation and give them protected time to reflect on the implementation effort, share lessons learned, and support one another's learning.
    m. 52: Promote network weaving
        i. Definition: Identify and build on existing high quality working relationships and networks within and outside the organization, organizational units, teams, etc. to promote information sharing, collaborative problem-solving, and a shared vision/goal related to implementing the innovation.
            1. Look for: Interdisciplinary team
    n. 57: Recruit, designate, and train for leadership
        i. Definition: Recruit, designate, and train leaders for the change effort.
            1. Look for: training of leaders (vs. strategy #35)
    o. 64: Use advisory boards and workgroups
        i. Definition: Create and engage a formal group of multiple kinds of stakeholders to provide input and advice on implementation efforts and to elicit recommendations for improvements.
    p. 65: Use an implementation advisor
        i. Definition: Seek guidance from experts in implementation.
    q. 72: Visit other sites
        i. Definition: Visit sites where a similar implementation effort has been considered successful.

5. <u>training and education of stakeholders (11)</u>
    a. 15: Conduct educational meetings
        i. Definition: Hold meetings targeted toward different stakeholder groups (e.g., providers, administrators, other organizational stakeholders, and community, patient/consumer, and family stakeholders) to teach them about the clinical innovation.
    b. 16: Conduct educational outreach visits
        i. Definition: Have a trained person meet with providers in their practice settings to educate providers about the clinical innovation with the intent of changing the provider's practice.
    c. 19: Conduct ongoing training
        i. Definition: Plan for and conduct training in the clinical innovation in an ongoing way.
    d. 20: Create a learning collaborative
        i. Definition: Facilitate the formation of groups of providers or provider organizations and foster a collaborative learning environment to improve implementation of the clinical innovation.
    e. 29: Develop educational materials
        i. Definition: Develop and format manuals, toolkits, and other supporting materials in ways that make it easier for stakeholders to learn about the

© 2022 Tewari P et al. *JAMA Network Open.*

innovation and for clinicians to learn how to deliver the clinical innovation.

f. 31: Distribute educational materials
    i. Definition: Distribute educational materials (including guidelines, manuals and toolkits) in person, by mail, and/or electronically.
g. 43: Make training dynamic
    i. Definition: Vary the information delivery methods to cater to different learning styles work contexts, and shape the training in the innovation to be interactive.
h. 55: Provide ongoing consultation
    i. Definition: Provide ongoing consultation with one or more experts in the strategies used to support implementing the innovation.
i. 60: Shadow other experts
    i. Definition: Provide ways for key individuals to directly observe experienced people engage with or use the targeted practice change/innovation.
j. 71: Use train-the-trainer strategies
    i. Definition: Train designated clinicians or organizations to train others in the clinical innovation.
k. 73: Work with educational institutions
    i. Definition: Encourage educational institutions to train clinicians in the innovation.

6. <u>support of clinicians (5)</u>
    a. 21: Create new clinical teams
        i. Definition: Change who serves on the clinical team, adding different disciplines and different skills to make it more likely that the clinical innovation is delivered (or is more successfully delivered).
    b. 30: Develop resource sharing agreements
        i. Definition: Develop partnerships with organizations that have resources needed to implement the innovation
    c. 32: Facilitate relay of clinical data to providers
        i. Definition: Provide as close to real-time data as possible about key measures of process/outcomes using integrated modes/channels of communication in a way that promotes use of the targeted innovation.
    d. 58: Remind clinicians
        i. Definition: Develop reminder systems designed to help clinicians to recall information and/or prompt them to use the clinical innovation.
    e. 59: Revise professional roles
        i. Definition: Shift and revise roles among professionals who provide care, and redesign job characteristics

© 2022 Tewari P et al. *JAMA Network Open.*

7. <u>engagement with consumers (5)</u>
    a. 37: Increase demand
        i. Definition: Attempt to influence the market for the clinical innovation to increase competition intensity and to increase the maturity of the market for the clinical innovation.
    b. 39: Intervene with patients/consumers to enhance uptake and adherence
        i. Definition: Develop strategies with patients to encourage and problem solve around adherence.
    c. 41: Involve patients/consumers and family members
        i. Definition: Engage or include patients/consumers and families in the implementation effort.
    d. 50: Prepare patients/consumers to be active participants
        i. Definition: Prepare patients/consumers to be active in their care, to ask questions, and specifically to inquire about care guidelines, the evidence behind clinical decisions, or about available evidence-supported treatments.
    e. 69: Use mass media
        i. Definition: Use media to reach large numbers of people to spread the word about the clinical innovation.

8. <u>use of financial strategies (9)</u>
    a. 1: Access new funding
        i. Definition: Access new or existing money to facilitate the implementation.
    b. 2: Alter incentive/allowance structures
        i. Definition: Work to incentivize the adoption and implementation of the clinical innovation.
    c. 3: Alter patient/consumer fees
        i. Definition: Create fee structures where patients/consumers pay less for preferred treatments (the clinical innovation) and more for less-preferred treatments.
    d. 28: Develop disincentives
        i. Definition: Provide financial disincentives for failure to implement or use the clinical innovations.
    e. 34: Fund and contract for the clinical innovation
        i. Definition: Governments and other payers of services issue requests for proposals to deliver the innovation, use contracting processes to motivate providers to deliver the clinical innovation, and develop new funding formulas that make it more likely that providers will deliver the innovation.
    f. 42: Make billing easier
        i. Definition: Make it easier to bill for the clinical innovation.
    g. 49: Place innovation on fee for service lists/formularies

© 2022 Tewari P et al. *JAMA Network Open.*

        i. Definition: Work to place the clinical innovation on lists of actions for which providers can be reimbursed (e.g., a drug is placed on a formulary, a procedure is now reimbursable).
    h. 66: Use capitated payments
        i. Definition: Pay providers or care systems a set amount per patient/consumer for delivering clinical care.
    i. 70: Use other payment schemes
        i. Definition: Introduce payment approaches (in a catch-all category).

9. <u>Change of infrastructure (8)</u>
    a. 9: Change accreditation or membership requirements
        i. Definition: Strive to alter accreditation standards so that they require or encourage use of the clinical innovation. Work to alter membership organization requirements so that those who want to affiliate with the organization are encouraged or required to use the clinical innovation.
    b. 10: Change liability laws
        i. Definition: Participate in liability reform efforts that make clinicians more willing to deliver the clinical innovation.
    c. 11: Change physical structure and equipment
        i. Definition: Evaluate current configurations and adapt, as needed, the physical structure and/or equipment (e.g., changing the layout of a room, adding equipment) to best accommodate the targeted innovation.
    d. 12: Change record systems
        i. Definition: Change records systems to allow better assessment of implementation or clinical outcomes.
    e. 13: Change service sites
        i. Definition: Change the location of clinical service sites to increase access.
    f. 22: Create or change credentialing and/or licensure standards
        i. Definition: Create an organization that certifies clinicians in the innovation or encourage an existing organization to do so. Change governmental professional certification or licensure requirements to include delivering the innovation. Work to alter continuing education requirements to shape professional practice toward the innovation.
    g. 44: Mandate change
        i. Definition: Have leadership declare the priority of the innovation and their determination to have it implemented.
    h. 62: Start a dissemination organization
        i. Definition: Identify or start a separate organization that is responsible for disseminating the clinical innovation. It could be a for-profit or non-profit organization.

© 2022 Tewari P et al. *JAMA Network Open.*
